# Accelerated Partial Breast Irradiation Delivered with Helical Tomotherapy: Dosimetry and Volumetric Predictors of Ipsilateral Breast Dose

**DOI:** 10.3390/cancers18132122

**Published:** 2026-06-30

**Authors:** Eva Yu-Hsuan Chuang, Chen-Hsi Hsieh, Pei-Wei Shueng, Chen-Xiong Hsu, Deng-Yu Kuo, Yueh-Feng Lu, Hsin-Pei Yeh, Pei-Yu Hou

**Affiliations:** 1 Division of Radiation Oncology, Department of Radiology, Far Eastern Memorial Hospital, New Taipei City 220, Taiwan; evachuang1219@gmail.com (E.Y.-H.C.); femh87690@femh.org.tw (C.-H.H.);; 2 School of Medicine, College of Medicine, National Yang Ming Chiao Tung University, Taipei 112, Taiwan; 3 Department of Computer Science and Engineering, Yuan Ze University, Taoyuan 320, Taiwan

**Keywords:** accelerated partial breast irradiation (APBI), helical tomotherapy (HT), volumetric predictors, ipsilateral breast dose, V15Gy

## Abstract

**Background/Objectives**: To evaluate the dosimetric performance and early clinical outcomes of five-fraction accelerated partial breast irradiation (APBI) delivered with helical tomotherapy (HT), and to identify volumetric parameters associated with ipsilateral breast dose exposure. **Methods**: We retrospectively reviewed early-stage breast cancer patients treated with APBI using HT (30 Gy in five fractions) between January 2024 and June 2025. Dosimetric parameters, acute toxicity, and early oncologic outcomes were analyzed. Correlation and receiver operating characteristic (ROC) analyses were performed to assess the association between volumetric parameters and ipsilateral breast V15Gy. **Results**: Sixteen patients were included with a median follow-up of 17.7 months. Mean PTV volume was 179.1 ± 53.9 mL, and mean PTV V95% was 98.0 ± 2.7%. Organ-at-risk sparing was favorable, with a mean heart dose of 0.82 ± 0.59 Gy and ipsilateral lung mean dose of 2.91 ± 0.45 Gy. The PTV-to-whole-breast volume ratio showed a significant correlation with ipsilateral breast V15Gy (β = 30.06, R^2^ = 0.34, *p* = 0.019) with a cutoff value of approximately 0.33 to predict ipsilateral breast V15Gy ≥ 50%. Acute toxicity was mild with no grade ≥ 2 dermatitis observed. **Conclusions**: Five-fraction APBI delivered with HT is dosimetrically feasible with favorable early toxicity profiles. The PTV-to-whole-breast volume ratio may serve as a simple and clinically applicable planning metric for anticipating ipsilateral breast dose.

## Simple Summary

Modern breast radiotherapy is moving toward shorter treatment schedules with greater precision. In this study, we evaluated accelerated partial breast irradiation treated with helical tomotherapy in five treatments. We found that it provides accurate dose delivery with low toxicity. We also identified a simple measurement—the ratio between the treated volume and the whole breast—that can help predict whether treatment plans will possibly meet dose limits. This practical indicator may support better treatment planning and improve the personalization of breast radiotherapy.

## 1. Introduction

Breast cancer is known to be the leading cause of global cancer incidence and cancer mortality [1]. Although breast-conservative surgery (BCS) and adjuvant radiation therapy (RT) remains the cornerstone treatment of early-stage breast cancer, efforts toward the de-escalation of local therapy have demonstrated comparable outcomes owing to advances in systemic therapy and molecular profiling [2,3]. Trials focusing on reducing treatment volume and shortening overall treatment time including NSABP B-39/RTOG 0413, RAPID, IMPORT LOW and the GEC-ESTRO have ultimately led to the endorsement of accelerated partial breast irradiation (APBI) as a standard treatment option for early-stage invasive breast cancer or ductal carcinoma in situ patients with favorable characteristics [4,5,6,7,8].

More recently, particularly in response to the COVID-19 pandemic, ultra-hypofractionated APBI schedules have gained increasing attention. The Florence randomized phase III trial first demonstrated that APBI delivered with intensity-modulated radiotherapy (IMRT) at 30 Gy in five fractions can achieve non-inferior long-term local control compared with conventional whole-breast irradiation (WBI), while significantly reducing treatment-related toxicity and improving cosmetic outcomes [9,10]. The study group also updated their results and dose constraints for plan optimization after shifting treatment techniques from IMRT to volumetric modulated arc therapy (VMAT) [11].

Despite the widespread adoption of modern external beam techniques for APBI, most published experiences have utilized static IMRT, VMAT or interstitial brachytherapy, while data regarding APBI delivered using helical tomotherapy (HT) remain limited. In ultra-hypofractionated regimens, where geometric uncertainty may have a proportionally greater impact on delivered dose, HT could offer potential advantages due to its highly conformal dose distributions and robust daily image guidance. In addition, patient-specific anatomical and volumetric factors may also play a critical role in determining dose distribution in highly conformal treatment settings, and identifying such factors could help guide patient selection and improve individualized treatment planning. Reports describing APBI delivered with helical tomotherapy remain scarce, comprising mainly small dosimetric or single-arm experiences [12,13].

With the increasing use of hypo-fractionated and stereotactic approaches, achieving optimal target coverage while minimizing dose to the ipsilateral breast tissue has become increasingly important since excessive dose spillage within the breast has been associated with inferior cosmetic outcomes and an increased risk of radiation-induced toxicity [14,15].

Given our institution’s extensive experience on tomotherapy platforms, we started utilizing HT for five-fraction APBI regimens since 2024. In this study, we aimed to evaluate the dosimetry and early clinical outcomes of HT-based APBI, and further explore volumetric parameters associated with ipsilateral breast dose.

## 2. Materials and Methods

### 2.1. Patient Selection and Data Collection

We retrospectively reviewed early-stage breast cancer patients treated with helical tomotherapy at our institution between January 2024 and June 2025. Eligible patients underwent breast-conserving surgery and received adjuvant APBI delivered in five fractions. Patient selection for APBI was based on our institutional criteria (Supplementary Table S1) which is consistent with the contemporary ASTRO guideline recommendations [8]. Specifically, eligible patients had unifocal, early-stage invasive ductal carcinoma, mucinous carcinoma or ductal carcinoma in situ (DCIS) with a tumor size ≤ 2 cm, histological grading 1–2, estrogen receptor (ER)-positive, node-negative (cN0/pN0) disease, and negative surgical margins. An age threshold of ≥40 years was applied in accordance with the 2024 ASTRO partial breast irradiation guideline [8], which supports PBI for selected patients aged ≥40 years with favorable features; for the 40–49-year subgroup, this represents a conditional recommendation based on moderate-quality evidence. Although the American Brachytherapy Society consensus statement [16] lists patients aged ≥45 years as appropriate candidates, our threshold reflects the more recent ASTRO guideline and the inclusion of substantial proportions of patients aged <50 years in pivotal PBI randomized trials [4,7,9].

This study received approval from the Human Experimentation Committee of Far Eastern Memorial Hospital (FEMH-114175E).

### 2.2. Simulation and Target Delineation

All patients were simulated in supine position under free-breathing with a 2.5 mm slice thickness (Discovery CT590 RT, GE Healthcare, Chicago, IL, USA) while customized vacuum bags were applicated for immobilization. For volume definitions, we refer to the APBI-IMRT-Florence trial by adding a uniform 1–1.5 cm, three-dimensional (3D) margin around the surgical clips for creation of clinical target volume (CTV). Any marks of change in the surrounding tissue architecture were also incorporated into the CTV, similar to the way we contour surgical beds. The CTV was limited to 4 mm from skin surface and excluded the chest wall and pectoralis muscles. A 5 mm expansion from the CTV was used to generate the planning target volume (PTV) to overcome setup uncertainties and respiratory motion. Organs at risk (OAR) included the heart, ipsilateral and contralateral lungs, ipsilateral and contralateral breasts, thyroid and whole body outside the PTV for assessment of dose spillage.

### 2.3. Treatment Planning and Delivery

All treatment plans were designed using the TomoTherapy Hi Art Planning system (version 5.1.3, Tomotherapy, Inc., Madison, WI, USA). The prescribed dose was 30 Gy in five fractions, delivered on non-consecutive days. Planning objectives for PTV coverage was to ensure at least 95% of PTV received 95% of the prescribed dose. Hot spots within the target were limited, constraining the volume receiving more than 105% of the prescription dose to less than 5% of the PTV. Laterality-specific heart constraints were applied as V1.5Gy ≤ 10% for right-sided tumors and heart V4.5Gy ≤ 10% for left-sided tumors. Due to the correlation of ipsilateral breast V50% with overall cosmesis, fat necrosis, fibrosis and induration shown in previous studies [17,18,19], we followed the constraints applied by the Florence study group by keeping ipsilateral breast V15Gy < 50–60%. Other dose constraints for lung, breast, and dose spillage outside the PTV were also enforced during optimization. Daily megavoltage computed tomography (MVCT) image guidance was used for all fractions to ensure accurate target localization.

### 2.4. Data Collection and Analysis

Dosimetric parameters were extracted from approved treatment plans, including PTV coverage, heart dose metrics, lung dose metrics, contralateral breast maximum dose and high-dose spillage outside the PTV (maximum dose outside PTV and body V32.1–33 Gy outside PTV). Patient characteristics were summarized with descriptive statistics. Categorical variables are presented as frequencies and percentages, whereas continuous variables are expressed as means with standard deviations (SDs). Acute toxicity was assessed using CTCAE version 5.0, focusing on acute dermatitis. Follow-up data were collected to describe early oncologic outcomes, including local control and survival status.

To explore the determinants of dose exposure to the ipsilateral breast (defined as ipsilateral breast excluding the CTV), volumetric parameters were evaluated, including absolute target volumes (CTV and PTV) and their ratios to ipsilateral whole-breast volume (CTV-to-whole-breast and PTV-to-whole-breast ratios). Relative parameters including tumor size and body mass index (BMI) were also evaluated. Associations between these variables and ipsilateral breast V15Gy (%) were assessed using linear regression analysis, with β coefficients and coefficients of determination (R^2^) reported to quantify effect size and explanatory power. The normality of continuous variables was examined using the Shapiro–Wilk test before analysis. Owing to the limited sample size, only univariable linear regression models containing a single predictor were fitted, and no multivariable modeling was performed, in order to avoid overfitting.

In addition, to assess the ability of these parameters to discriminate patients with higher dose exposure, ipsilateral breast V15Gy was dichotomized at 50% (V15Gy ≥ 50% vs. <50%), and receiver operating characteristic (ROC) curve analysis was performed. The area under the ROC curve (AUC) was used to quantify discriminatory performance. Optimal cutoff values were determined using the Youden index. To further reduce the risk of overfitting in this small cohort, each ROC analysis evaluated a single predictor, and no composite or multivariable classifiers were derived; accordingly, all regression and ROC results were interpreted as exploratory and hypothesis-generating.

All analyses were performed using SPSS (version 21.0; IBM, Armonk, NY, USA) and R (RStudio; R version 4.x). All statistical tests were two-sided, and *p* values < 0.05 were considered statistically significant.

## 3. Results

### 3.1. Patient Characteristics and Treatment Overview

Sixteen patients treated with APBI at our institution using helical tomotherapy were included in this analysis, as summarized in Table 1. The median follow-up duration was 17.7 months (mean 17.4 months, range 12.1–25.2 months) and the median age at treatment was 54 years (range 42–79 years). Tumors were located in the right breast in seven patients (43.8%) and left breast in nine patients (56.3%). The mean CTV and PTV were 74.4 ± 38.3 mL and 179.1 ± 52.2 mL, respectively. All patients received 30 Gy in five fractions following breast-conserving surgery and were treated on non-consecutive days. No treatment interruptions occurred, and all patients completed the prescribed course as planned.

### 3.2. Target Coverage and Plan Quality

**Figure 1:**
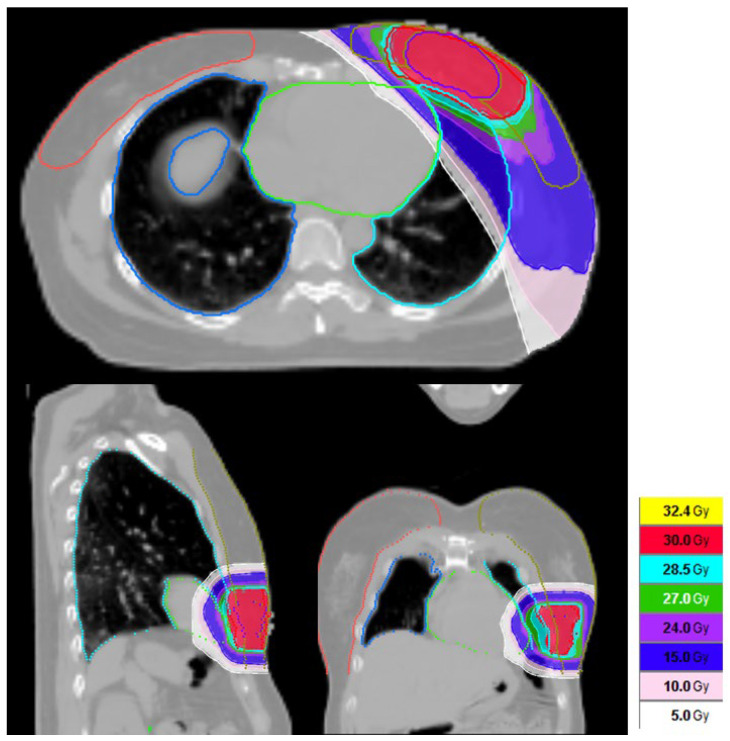
Representative left-sided APBI plan delivered with helical tomotherapy. Red indicates the prescription dose (30 Gy, 100%), green and blue represent intermediate dose levels. This APBI plan shows conformal target coverage with minimal cardiac and pulmonary exposure.

Target coverage was generally satisfactory, with a mean PTV V95% of 98.0 ± 2.7%. Thirteen of sixteen patients (81.3%) achieved a PTV V95% ≥ 95%. OAR sparing was consistently favorable. The mean heart dose across the APBI cohort was 0.82 ± 0.59 Gy, with a mean whole-lung dose of 1.62 ± 0.38 Gy and an ipsilateral lung mean dose of 2.91 ± 0.45 Gy. The volume of low-dose exposure to the lung was also limited, with average ipsilateral lung V9Gy of 7.9% and contralateral lung V3Gy of 1.76%. All patients met laterality-specific heart constraints, defined as heart V1.5Gy ≤ 10% for right-sided tumors and heart V4.5Gy ≤ 10% for left-sided tumors. A representative treatment plan is shown in Figure 1, demonstrating conformal target coverage with rapid dose fall-off in a left-sided APBI case.

High-dose spillage outside the target was minimal. The volume of body tissue receiving 32.1–33 Gy outside the PTV was negligible in all cases, with a maximum of 0.07 cc, while maximum dose outside the PTV were all within predefined limits of 33–33.6 Gy (Table 2). The contralateral breast maximum dose was generally low (mean 1.78 ± 2.42 Gy), although one case exceeded the recommended threshold due to target proximity. The relationship between tumor bed location and contralateral breast Dmax was also explored, which showed no statistically significant association.

### 3.3. Ipsilateral Breast Dose and Volumetric Correlations

**Figure 2:**
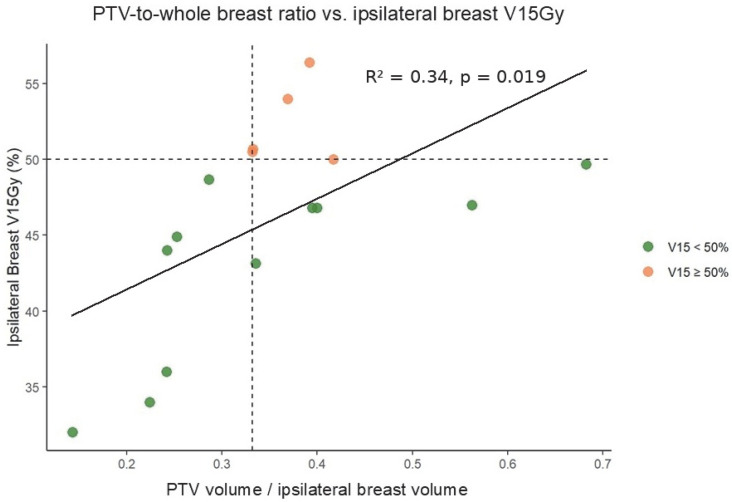
Scatter plot showing the relationship between PTV-based volumetric ratio and ipsilateral breast V15Gy. The vertical dashed line represents the optimal cutoff derived from ROC analysis, and the horizontal dashed line indicates the clinical threshold of V15Gy = 50%. Cases are colored according to whether the ipsilateral breast V15Gy was below or above 50%. The solid line represents the linear regression fit (R^2^ = 0.34, *p* = 0.019).

To further characterize determinants of ipsilateral breast dose exposure, volumetric parameters were evaluated. The PTV-to-whole-breast volume ratio demonstrated a significant positive association with ipsilateral breast V15Gy (β = 30.06, R^2^ = 0.34, *p* = 0.019), as shown in Figure 2, whereas the CTV-to-breast volume ratio showed a weaker and non-significant association (β = 34.79, R^2^ = 0.10, *p* = 0.235) (Supplementary Figure S1). Absolute volumetric parameters, including PTV volume, CTV volume, tumor size, and BMI, showed limited explanatory power and no statistically significant association with ipsilateral breast V15Gy (Table 3).

When ipsilateral breast V15Gy was dichotomized at 50%, ROC analysis demonstrated modest discriminatory ability for both volumetric ratios. The PTV-to-whole-breast volume ratio showed limited discrimination, with an optimal cutoff value of 0.33 (AUC = 0.64), yielding a sensitivity of 100% and a specificity of 54.5% for predicting V15Gy ≥50% (Supplementary Figure S2A). In comparison, the CTV-to-whole-breast volume ratio demonstrated similar but slightly higher discriminatory performance, with an AUC of 0.69 (Supplementary Figure S2B). Other evaluated parameters showed lower discriminatory performance.

### 3.4. Acute Toxicity and Early Clinical Outcomes

Acute toxicity was mild. Acute dermatitis was limited to Grade 0–1 in all patients, with no Grade ≥ 2 skin reactions observed. In addition to dermatitis, no Grade ≥ 2 breast pain, breast edema, seroma formation, hyperpigmentation, or fatigue was observed; any such symptoms, when present, were Grade 1 and self-limiting. No ipsilateral breast pain or induration requiring intervention, radiation pneumonitis, or cardiac events were recorded during the early follow-up period. No local recurrence or disease-related death was observed at the latest follow-up. The medications taken by each patient, including guideline-concordant adjuvant systemic and/or endocrine therapy and treatments for underlying comorbidities, were cataloged for all patients. No apparent association was observed between these medications and the mild acute toxicity recorded.

## 4. Discussion

### 4.1. Feasibility and Comparison with Prior APBI Studies

Our study demonstrates that five-fraction APBI delivered with HT can achieve satisfactory dosimetric performance according to ASTRO clinical practice guidelines [8]. In this real-world clinical setting, we observed consistently low cardiac and pulmonary exposure, minimal high-dose spillage, and minimal acute toxicity. Despite concerns regarding the “low-dose bath” inherent to rotational delivery techniques, our results indicate that careful planning can effectively constrain both low- and high-dose exposure to critical organs while maintaining adequate target coverage. These observations are in keeping with the maturing evidence base supporting ultra-hypofractionated APBI as a standard option for suitable patients [20].

The observed mean heart dose of 0.82 Gy and ipsilateral mean lung dose of 2.91 Gy are comparable with the reported updates of the APBI-Florence-IMRT trial using VMAT technique [10,11]. With a median follow-up of 4.5 years, they demonstrated no Grade > 1 acute or late toxicities and excellent cosmetic outcomes. In our institution, the CTV is created using a 1–1.5 cm expansion from the surgical clips similar to the Florence protocol but modified to include any change in the surrounding tissue architecture. The resulting PTV volume was slightly larger (mean: 179.1 ± 52.2 cm^3^) compared to the updated Florence study (mean PTV volume: 139 ± 48 cm^3^). Nevertheless, low heart and lung mean doses were achieved in our study, which highlights the strength of HT in OAR sparing. All patients met laterality-specific cardiac dose (V1.5 and V4.5) constraints, further supporting the feasibility of heart sparing on this platform. This is particularly relevant given the increasing emphasis on minimizing even low cardiac doses due to their potential long-term impact on cardiovascular morbidity [21,22].

### 4.2. High-Dose Spillage and Contralateral Breast Dose

Another notable result of our APBI-tomotherapy cohort is the control of high-dose spillage outside the PTV. All cases met stringent constraints for dose outside the target, with nearly negligible volumes receiving 32.1–33 Gy dose beyond the PTV. This is clinically meaningful, as excessive high-dose spillage has been shown to increase risks of fibrosis, fat necrosis, and suboptimal cosmetic outcomes [23,24]. In an exploratory analysis, we compared contralateral breast maximum dose in different tumor bed locations grouped as outer versus inner or central quadrants. Although no statistical significance was reached, inner or central tumors demonstrated a tendency toward higher contralateral breast dose. We found a single outlier case, in which the contralateral breast maximum dose exceeded the recommended constraint, that corresponded to an upper–inner quadrant tumor bed located close to the midline. Although the absolute dose remained modest, even low-level contralateral exposure is worth minimizing because of the recognized, dose-dependent risk of radiation-induced second primary breast cancer, particularly in younger patients with longer life expectancy [25,26]. This case underscores the value of explicitly reviewing contralateral breast dose during planning for medially located targets; conversely, the consistently low doses achieved in the remaining cohort suggest that the contralateral secondary-malignancy risk associated with this technique is expected to be low. Furthermore, sensitivity analysis excluding this case did not substantially alter the overall findings. This indicates that the tumor bed size and geometry, rather than quadrant classification alone, may contribute to contralateral dose spillage in APBI delivered with rotational techniques (Supplementary Figure S3).

### 4.3. Clinical Relevance of Volumetric Parameters and Ipsilateral Breast Dose

Despite favorable overall dosimetry outcomes, two issues merit further consideration. First, PTV V95% fell below 95% in a minority of patients, typically in cases where the target was adjacent to the skin or chest wall. This reflects the inherent trade-off between target coverage and the protection of superficial normal tissues in APBI. Second, ipsilateral breast V15Gy exceeded the recommended threshold in approximately one-third of patients. To further explore the determinants of ipsilateral breast dose exposure, we evaluated volumetric parameters based on both target size and relative breast volume. The PTV-to-whole-breast volume ratio demonstrated a consistent and statistically significant association with ipsilateral breast V15Gy, whereas the CTV-to-whole-breast volume ratio showed a weaker and non-significant association in linear regression analysis.

Interestingly, the performed ROC analysis showed that the CTV-to-whole-breast volume ratio yielded a numerically higher AUC (0.69) compared with the PTV-to-whole-breast volume ratio (0.64). This discrepancy likely reflects the small sample size and variability of the dataset, as AUC represents discriminatory ability rather than the strength or consistency of association. Therefore, a higher AUC does not necessarily indicate a more reliable or clinically applicable predictor. From a practical standpoint, the PTV-to-whole-breast volume ratio may represent a more robust parameter. It incorporates the setup margin and therefore better reflects the actual irradiated volume. The identified threshold of approximately 0.33 may serve as a useful feasibility indicator. It helps flag patients in whom achieving the recommended ipsilateral breast V15Gy constraint is more challenging. This result is also broadly consistent with the correlation found in the updated APBI-IMRT-Florence trial using VMAT technique [11]. Notably, mammographic density and breast composition vary across different racial and ethnic groups and may influence achievable dose distributions. Our cohort consisted exclusively of Asian patients, who tend to have smaller, denser breasts [27] and are under-represented in the APBI literature. The present data therefore provide dosimetric evidence for this specific population, in whom the ipsilateral breast V15Gy constraint might appear to be even more challenging. Validation in more ethnically diverse cohorts is warranted to confirm the generalizability of the proposed PTV-to-whole-breast volume ratio.

In routine practice, this parameter can be calculated immediately after contouring and before optimization begins. Cases with a ratio approaching or exceeding 0.33 may be flagged as potentially difficult for meeting ipsilateral breast V15Gy constraints. In such situations, planners may consider prioritizing ipsilateral breast dose reduction earlier in the optimization process, reviewing target margin appropriateness, and carefully balancing PTV coverage against superficial tissue sparing. This parameter may also help identify cases in which additional planning iterations or alternative delivery techniques should be considered. Importantly, it should be viewed as a planning aid rather than a strict selection criterion, guiding optimization strategies rather than excluding patients from APBI. Automated and knowledge-based planning approaches may further facilitate the consistent achievement of ipsilateral breast constraints in such challenging cases [28].

### 4.4. Strengths and Limitations

To our knowledge, this is the first study specifically focusing on five-fraction accelerated partial breast irradiation delivered with helical tomotherapy in patients with early-stage breast cancer. All patients completed treatment without interruption, and no Grade ≥ 2 acute dermatitis was observed. Although follow-up remains relatively short, the absence of early local recurrence and serious toxicity is reassuring and aligns with existing APBI literature [29]. A major strength of our present study is its real-world clinical setting, reflecting routine treatment planning and delivery rather than highly controlled, protocol-driven conditions. The use of a single treatment platform with consistent planning objectives and daily image guidance enabled a focused evaluation of dosimetric performance and dose–volume relationships inherent to APBI delivered with HT. Furthermore, the assessment of volumetric parameters, particularly the PTV-to-whole-breast volume ratio, offers a simple and reproducible parameter that may support plan evaluation in clinical practice. Our early outcomes are consistent with the contemporary five-fraction APBI series reporting low toxicity and favorable cosmesis [30,31,32,33], as well as with prior tomotherapy-based partial breast experiences [12,13]. Importantly, the primary endpoints of this study are dosimetric and are fully defined at the time of treatment planning and delivery; they are therefore mature and independent of the duration of clinical follow-up, so the present follow-up is adequate to address the dosimetric objectives of this study. The extended clinical follow-up (median 17.7 months), with all patients exceeding one year and without local recurrence or serious toxicity, further supports the favorable early safety of this approach.

Several limitations should be acknowledged. First, the retrospective nature and limited sample size reflect a relatively low volume of helical tomotherapy-based APBI from a single institution which restricts the statistical power and generalizability of the findings; the present results should therefore be regarded as exploratory and hypothesis-generating, and we are committed to validating them in larger, ideally multi-institutional and multi-ethnic cohorts. Second, follow-up duration remains short, precluding robust evaluation of late toxicity, cosmetic outcomes, and long-term local control. Third, although this study focused on tomotherapy-specific characteristics, direct comparisons with other delivery techniques such as VMAT or 3D conformal radiotherapy were not performed. In summary, our findings support helical tomotherapy as a viable and effective platform for APBI delivery. Future studies should be focused on refining planning parameters, identifying and validating predictors of suboptimal dosimetry, and reporting long-term results.

## 5. Conclusions

Five-fraction APBI delivered with helical tomotherapy is feasible in the treatment of early-stage breast cancer with favorable dosimetric and early toxicity outcomes. The PTV-to-whole-breast volume ratio may serve as a simple and practical planning metric for predicting ipsilateral breast V15Gy. Longer follow-up and further validation in larger cohorts are warranted.

## Figures and Tables

**Table 1:** Patient characteristics and treatment outcomes.

Characteristics	Value (*N* = 16)
Age, years, median (range)	54 (42–79)
BMI, mean ± SD	24.36 ± 3.86
Laterality, *n* (%)	
Right	7 (43.8%)
Left	9 (56.3%)
Histology, *n* (%)	
Invasive ductal carcinoma (IDC)	8 (50.0%)
Ductal carcinoma in situ (DCIS)	7 (43.8%)
Mucinous carcinoma	1 (6.2%)
Tumor grade, *n* (%)	
Grade 1	7 (43.8%)
Grade 2	9 (56.3%)
ER-positive, *n* (%)	16 (100.0%)
Tumor size (cm), mean ± SD	0.98 ± 0.49
Tumor bed location, *n* (%)	
Inner/Central	10 (62.5%)
Outer	6 (37.5%)
CTV volume (cc), mean ± SD	74.38 ± 38.30
PTV volume (cc), mean ± SD	179.12 ± 52.18
Follow-up months, median (range)	17.7 (12.1–25.2)
Acute dermatitis, *n* (%)	
Grade 0	6 (37.5%)
Grade 1	10 (62.5%)
Grade ≥ 2	0 (0%)
Local recurrence, *n* (%)	0 (0%)
Overall survival, *n* (%)	16 (100%)

Abbreviations: BMI, body mass index; CTV, clinical target volume; DCIS, ductal carcinoma in situ; ER, estrogen receptor; IDC, invasive ductal carcinoma; PTV, planning target volume; SD, standard deviation. Continuous variables are presented as mean ± SD or median (range); categorical variables as *n* (%).

**Table 2:** Dosimetric parameters and constraints.

Parameters	Mean ± SD	Range (Min–Max)	Constraints	Pass Rate
**Target coverage**				
PTV V95 (%)	98.0 ± 2.7	92.2–100.0	≥95%	81.3% (13/16)
PTV V105 (%)	1.8 ± 1.2	0.0–4.8	≤5%	93.8% (15/16)
**Heart**				
Heart mean dose (Gy)	0.82 ± 0.59	0.18–1.90	—	—
Heart V1.5Gy (%)—right-sided	2.1 ± 2.4	0.0–8.6	≤10%	100% (7/7)
Heart V4.5Gy (%)—left-sided	3.4 ± 3.1	0.0–9.2	≤10%	100% (9/9)
**Lung**				
Whole-lung mean dose (Gy)	1.62 ± 0.38	1.16–2.36	—	—
Ipsilateral lung mean dose (Gy)	2.91 ± 0.45	2.26–3.63	—	—
Ipsilateral lung V9Gy (%)	7.9 ± 3.3	6.7–14.8	<10%	93.8% (15/16)
Contralateral lung V3Gy (%)	1.76 ± 2.50	0.0–9.0	<5%	100% (16/16)
**Breast and dose spillage**				
Ipsilateral breast V28.5Gy (%)	19.6 ± 4.8	11.2–29.1	≤25%	93.8% (15/16)
Ipsilateral breast V15Gy (%)	46.2 ± 12.9	28.4–71.6	<50%	68.8% (11/16)
Contralateral breast Dmax (Gy)	1.78 ± 2.50	0.78–11.13	≤1.5 Gy	93.8% (15/16)
Body outside PTV V32.1–33 Gy (cc)	0.02 ± 0.08	0.0–0.30	≤2 cc	100% (16/16)
Body outside PTV Dmax (Gy)	32.4 ± 0.7	31.1–33.5	≤33.6 Gy	100% (16/16)

Constraints follow the APBI-IMRT-Florence protocol and institutional criteria. Abbreviations: Dmax, maximum dose; PTV, planning target volume; SD, standard deviation; V95/V105, percentage of the PTV receiving 95%/105% of the prescribed dose; VxGy, volume receiving x Gy. Pass rate denotes the number (and proportion) of plans meeting the stated constraint.

**Table 3:** Association of volumetric-related parameters with ipsilateral breast V15Gy.

Variables	β Coefficient	R^2^	*p*-Value	AUC (ROC)	Optimal Cutoff
PTV/breast volume ratio	30.06	0.336	0.019	0.636	0.332
CTV/breast volume ratio	34.79	0.099	0.235	0.691	0.158
PTV volume (mL)	0.028	0.049	0.408	0.618	153.2
CTV volume (mL)	0.002	0.000	0.974	0.655	84.6
Tumor size (cm)	0.82	0.005	0.806	0.260	0.35
BMI	−0.45	0.068	0.329	0.436	19.98

Univariable linear regression and receiver operating characteristic (ROC) analyses for predictors of ipsilateral breast V15Gy. Abbreviations: AUC, area under the ROC curve; BMI, body mass index; CTV, clinical target volume; PTV, planning target volume; R^2^, coefficient of determination; β, regression coefficient. The optimal cutoff was determined using the Youden index; statistical significance was set at *p* < 0.05.

## Data Availability

The data that support the findings of this study are available from the corresponding author upon reasonable request.

## Supplementary Materials

The following supporting information can be downloaded at https://www.mdpi.com/article/10.3390/cancers18132122/s1. Table S1: Criteria for APBI patient selection (adapted from ASTRO guidelines); Figure S1: Scatter plots showing the relationship between each volumetric predictor and ipsilateral breast V15Gy; Figure S2: ROC curves predicting ipsilateral breast V15Gy ≥50%: (A) PTV/whole-breast volume ratio; (B) CTV/whole-breast volume ratio; Figure S3: Contralateral breast Dmax stratified by tumor bed location (outlier excluded).

## Abbreviations

The following abbreviations are used in this manuscript:

3D	three-dimensional
APBI	accelerated partial breast irradiation
ASTRO	American Society for Radiation Oncology
AUC	area under the curve
BCS	breast-conserving surgery
BMI	body mass index
CT	computed tomography
CTCAE	Common Terminology Criteria for Adverse Events
CTV	clinical target volume
DCIS	ductal carcinoma in situ
Dmax	maximum dose
ER	estrogen receptor
GEC-ESTRO	Groupe Européen de Curiethérapie–European Society for Radiotherapy and Oncology
Gy	gray
HT	helical tomotherapy
IMRT	intensity-modulated radiotherapy
MVCT	megavoltage computed tomography
NSABP	National Surgical Adjuvant Breast and Bowel Project
OAR	organ(s) at risk
PTV	planning target volume
RAPID	Randomized Trial of Accelerated Partial Breast Irradiation
ROC	receiver operating characteristic
RT	radiation therapy
RTOG	Radiation Therapy Oncology Group
R^2^	coefficient of determination
SD	standard deviation
VMAT	volumetric modulated arc therapy
V15Gy	volume of an organ receiving 15 Gy
V95%	volume receiving 95% of the prescribed dose
WBI	whole-breast irradiation
