# Supplementary material for: Accelerated Partial Breast Irradiation Delivered with Helical Tomotherapy: Dosimetry and Volumetric Predictors of Ipsilateral Breast Dose
Source: Cancers (Basel). 2026 Jun 30;18(13):2122. doi: 10.3390/cancers18132122 (PMC13360138; doi:10.3390/cancers18132122)
Supplement: Supplementary file 1 [file cancers-18-02122-s001.zip › Supplementary Table S1.pdf]

Supplementary Table S1. Criteria for ABPI patient selection (adapted from ASTRO guidelines)

	**Suitable** (meets all criteria)	**Cautionary** (meets multiple criteria)	**Unsuitable** (meets any criteria)
**Age**	≥ 50	40-49	< 40
**Grade**	1-2	3	
**Tumor Size**	≤ 2 cm	2.1 – 3 cm	> 3 cm
**N stage, surgery**	pN0 (SNBx or ALND)	no nodal surgery	pN1-3
**Margins**	Negative	Close	Positive
**LVSI**	No	Limited/focal	Extensive
**ER status**	Positive	Negative	
**HER-2 status**			Positive but not receiving anti-HER2 therapy
**Centricity**	Unicentric	Multicentric	
**Histology**	Invasive ductal or favorable histology	Invasive lobular	
**BRCA1/2 mutation**	Negative		Positive
