# Supplementary figures and images for: Accelerated Partial Breast Irradiation Delivered with Helical Tomotherapy: Dosimetry and Volumetric Predictors of Ipsilateral Breast Dose
Source: Cancers (Basel). 2026 Jun 30;18(13):2122. doi: 10.3390/cancers18132122 (PMC13360138; doi:10.3390/cancers18132122)

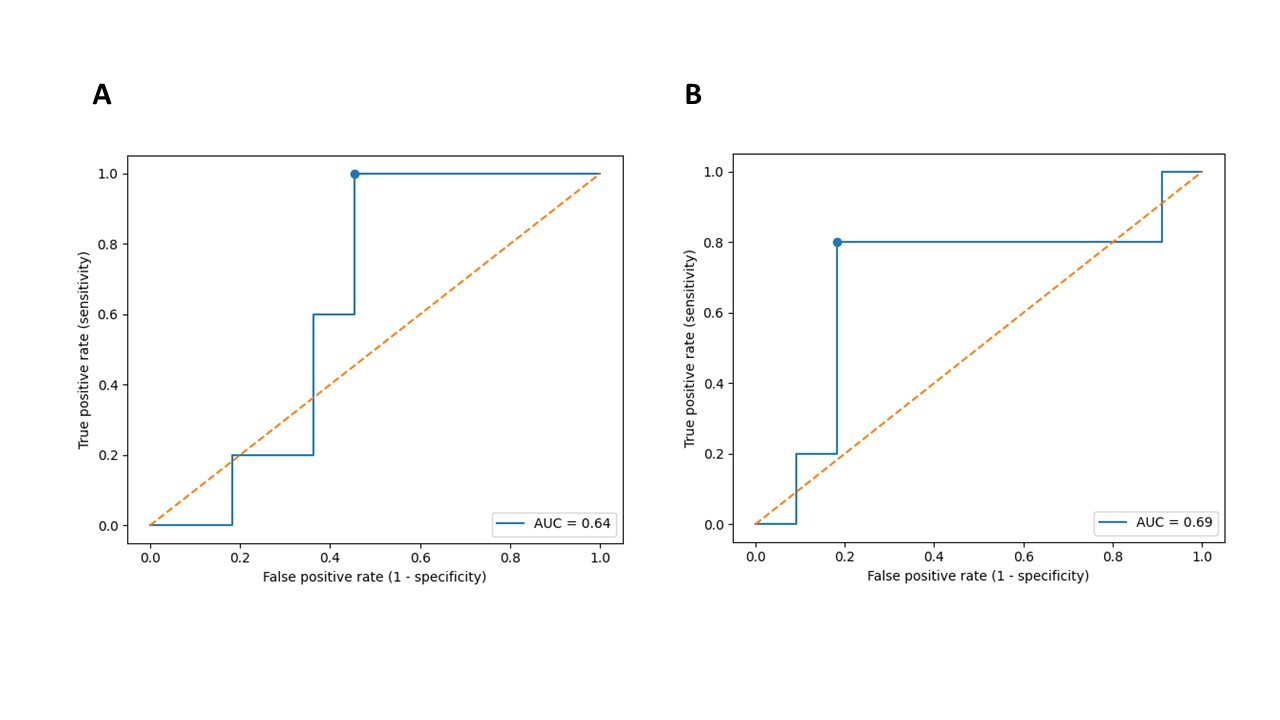

Supplement: Supplementary file 1 [file cancers-18-02122-s001.zip › Supplementary Figure S2 ROC curves predicting ipsilateral breast V15Gy ≥50%.jpg]

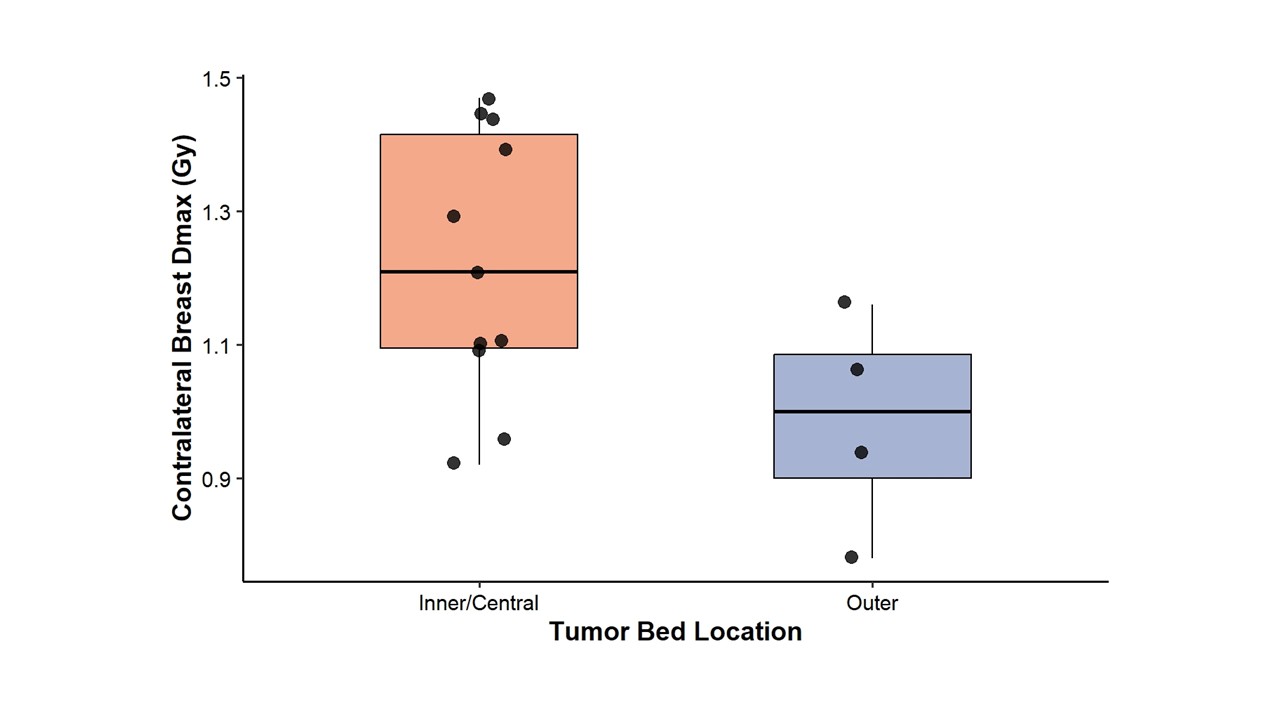

Supplement: Supplementary file 1 [file cancers-18-02122-s001.zip › Supplementary Figure S3 Contralateral breast Dmax stratified by tumor bed location (outlier excluded)..jpg]

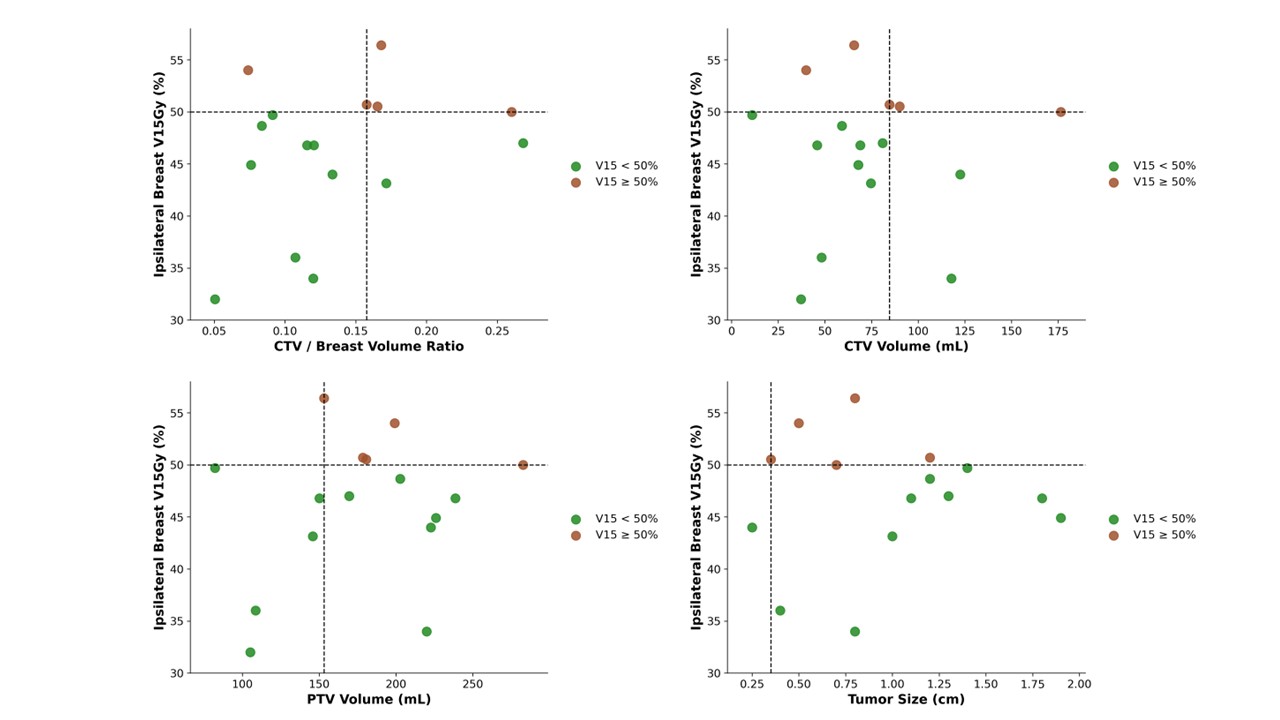

Supplement: Supplementary file 1 [file cancers-18-02122-s001.zip › Supplementary Figure S1 Scatter plots showing the relationship between each volumetric predictor and ipsilateral breast V15Gy.jpg]
